# Are There Differences in “Intelligence” Between Nonhuman Species? The Role of Contextual Variables

**DOI:** 10.3389/fpsyg.2020.02072

**Published:** 2020-08-21

**Authors:** Michael Colombo, Damian Scarf

**Affiliations:** Department of Psychology, University of Otago, Dunedin, New Zealand

**Keywords:** null hypothesis, contextual variables, Macphail, intelligence, species differences

## Abstract

We review evidence for Macphail’s (1982, 1985, 1987) *Null Hypothesis*, that nonhumans animals do not differ either qualitatively or quantitatively in their cognitive capacities. Our review supports the *Null Hypothesis* in so much as there are no qualitative differences among nonhuman vertebrate animals, and any observed differences along the qualitative dimension can be attributed to failures to account for contextual variables. We argue species do differ quantitatively, however, and that the main difference in “intelligence” among animals lies in the degree to which one must account for contextual variables.

## Macphail’s Claim

In the present case we should, then, conclude that there are no differences, either qualitative or quantitative, among vertebrates (excluding man; Macphail, 1985, p. 46).

In 1985, Macphail advocated the *Null Hypothesis* for animal intelligence which stated that there are no differences, either qualitative or quantitative, in intelligence across nonhuman species. Macphail later published his *Null Hypothesis* as a target article in *Behavioral and Brain Sciences* (Macphail, 1987). It is fair to say that the peer commentaries were generally negative. For example, Sternberg (1987, p. 680) stated that “Macphail has made a valiant but not wholly successful effort” while Elepfandt (1987, p. 662) commented with respect to the newly emerging study of vertebrate intelligence that “This new growth should not be stunted by narrow views or precipitate conclusions.” Perhaps the most scathing comment was lodged by Goldman-Rakic and Preuss (1987, p. 667) who stated that “Macphail’s ‘null hypothesis’ is merely the epitaph on the head stone of comparative cognition.”

Rather than stunting the growth of comparative cognition or becoming the epitaph on its headstone, in the more than three decades since the publication of *Vertebrate intelligence: the null hypothesis* (Macphail, 1985) there has been an explosion of research into the cognitive capacities of animals. Topics, such as *episodic memory*, *theory of mind*, and *planning for the future* were little investigated in 1985, whereas now they form the mainstay of animal cognition studies. And other topics such as the *representation of equivalence relations* (reflexivity, symmetry, and transitivity) have enjoyed a long research history and have continued to generate considerable insight into the mental abilities of nonhuman animals. In light of the wealth of data that has accumulated since Macphail (1985) published his *Null Hypothesis*, the aim of this article is to see whether it has stood the test of time: are there really no differences, qualitative or quantitative, in the cognitive abilities of vertebrate animals?

## Some Background Issues

In the present article, we review the current status of Macphail’s claim that there are no differences, either qualitative or quantitative, in intelligence across nonhuman vertebrate species. Many of the criticisms directed at Macphail (1987) concerned his use of the term “intelligence.” For example, Barlow (1987, p. 657) put it perfectly when he stated “because there is not yet any generally agreed upon definition of intelligence that enables a quantitative scale to be defined for it…it cannot justifiably be said that quantitative differences either do, or do not, exist.” We believe this is a fair criticism, and furthermore agree with Hodos (1987, p. 668) when he stated that “we should not become bogged down with a general intelligence concept for animals because its measurement is well beyond our grasp.”

Limitations in the definition of “intelligence” aside, the field of comparative cognition is about comparing the abilities of different animals in order to understand not only their capacities but also the evolution of the mental abilities of humans. Evaluating how animals differ in “intelligence,” however, may not be the best approach. Rather, we think a better approach is to concentrate on specific, definable, and measurable capacities that allow direct comparisons to be made between species. D’Amato and Salmon (1984, p. 149) put forward such a view with respect to comparing the cognitive abilities of different species when they said “how much simpler the task would be if we could identify a relatively small number of kernel cognitive capabilities that would allow us, through their measurement, to make reasonable statements about the cognitive potentials and capacities of various species.” To this extent, we focus on a set of such kernel cognitive abilities that have been the subject of extensive investigations across species: reflexivity, symmetry, transitivity, and serial-order behavior, as well as touch upon some more contemporary kernel cognitive abilities such as episodic memory and ToM.

Besides issues surrounding the use of the term “intelligence,” another caveat concerns our use of the term “cognition.” We use the term more for ease of exposition than necessarily to indicate that our animals are solving tasks using processes that go beyond, or are unexplained by, behavioral principles encompassed by operant and classical conditioning or associative processes. Effectively, our use of the term “cognition” is synonymous with Shettleworth’s (1998, p. 5) definition that it encompasses “the mechanisms by which animals acquire, process, store and act on information from the environment.”

A third caveat is that it is not our intention to compare the cognitive abilities of nonhuman animals with those of humans. Such comparisons have been dealt with extensively in a recent review (Penn et al., 2008). Rather, our aim is to compare the cognitive abilities of nonhuman animals and specifically, address the value of the *Null Hypothesis*. Similarly, because most research has been conducted on either apes, monkeys, rats, or birds, our comparisons are limited to these species. That said, these species offer a sufficient range of evolutionary independence, as well as differences in neuroanatomy and niches, to forestall any criticism that we failed to sample widely.

## The Role of Contextual Variables

In 1965, Bitterman advanced the idea of a contextual variable, a noncognitive factor that accounts for the differences in observed behavior between species. Speaking with respect of the inability of fish to display improvements in reversal learning on both spatial and visual task, Bitterman (1965, p. 95) stated that:

*“Another possibility is that the conditions under which the fish has been tested are to blame for its poor showing, that the difference in performance is to be traced not only to a difference in capability but also to an inequality in some contextual variable, such as sensory demand, motor demand, degree of hunger, or attractiveness of reward.”*

Bitterman (1965, p. 95) also foresaw the problem with the notion of contextual variables when he stated “Can we ever, then, rule out the possibility that a difference in performance of two different animals in such an experiment stems from a difference in some confounded contextual variable?” Macphail (1985, p. 39) revisited the notion of contextual variables in his paper and echoed the same concern when he stated that “There is no finite catalogue of potentially relevant contextual variables: how, therefore, could their effects be conclusively ruled out?”

While the concerns around the issue of contextual variables are reasonable, we believe contextual variables do lie on a continuum of importance and relevance. Although one might be justified in doing so, few would be tempted to argue that a difference in ability between species A and B was because the stimuli used in the experiment with species A were different in size to those used in the experiment with species B. On the other hand, an apparatus that prevents an animal from properly processing a stimulus would indeed be a valid appeal to a contextual variable. Indeed Macphail (1985) went on to conclude that the importance of contextual variables cannot be overlooked, and we fully subscribe to that view. As we will show in the current review, contextual variables often play a role in the outcome of whether an animal can display a certain ability.

We first focus on a set of cognitive capacities referred to as equivalence relations (reflexivity, symmetry, and transitivity). Although the idea of equivalence relations may not spark the notion of cognitive prowess, equivalence relations underlie a number of complex behaviors. According to Sidman (2018, p. 33), for example, equivalence relations play a central role “in making language such a powerful factor in our everyday social intercourse with each other.”

### Reflexivity

The first equivalence relation we explore is reflexivity known better in the animal cognition literature as the “same-different” or “matching” concept. The task most frequently used to explore whether animals can form a matching concept is the simultaneous matching-to-sample (SMS) task. Although there are many variants, the basic procedure is very simple. An animal is shown a sample stimulus, for example, either a circle or vertical line geometric form. After responding to the sample stimulus two comparison stimuli appear on either side of the sample stimulus, one the same as the sample and the other different. The animal must respond to the comparison stimulus that is the same as the sample stimulus. In this example, from trial to trial, the sample alternates between the circle and vertical line stimuli.

An animal can solve a SMS task in one of three main ways (Skinner, 1950; Farthing and Opuda, 1974; Carter and Werner, 1978). One way is by learning each of the possible configurations of the sample and comparison stimuli. With two stimuli (A and B), and the stimuli arranged so that the sample stimulus appears in the center and the comparison stimuli appear on either side of the sample stimulus, there are four possible sample-comparison configurations, AAB, BAA, BBA, and ABB. According to the *configuration* view, the animal learns that the configurations AAB and BBA mean peck the left stimulus to obtain a reward, and the configurations ABB and BAA mean peck the right stimulus to obtain a reward. A second way to solve a SMS task is by learning a series of *stimulus-response associations* such as “if circle was the sample then press the circle comparison stimulus” and “if vertical line was the sample then press the vertical line comparison stimulus.” Finally, a third way to solve a SMS task is by learning a *generalized matching concept* such as “peck the comparison stimulus that matches the sample stimulus.” Solving the task by implementing a generalized matching concept is “a necessary consequence of reflexivity, which therefore conveys the notion of sameness” (Sidman et al., 1982, p. 24).

To untangle which of the three possible ways an animal may be solving a SMS task, a transfer test is conducted in which the subjects are presented with novel stimuli, such as red and green. There are a multitude of issues about the conditions that must prevail during the transfer test in order to infer solution by a matching concept. First, the “novel” stimuli must be truly novel in the sense that one should not be able to invoke the notion of stimulus generalization to account for the good transfer performance. In other words, if we train an animal with a circle and vertical line as the stimuli, and tested them with oval and tilted line, the good performance on the transfer test is more likely attributable to stimulus generalization than the application of a matching concept. To avoid the pitfall of stimulus generalization, the stimuli on the transfer test should be completely different (i.e., orthogonal) to the training stimuli. In our example, the transfer stimuli of red and green are orthogonal to the training stimuli of a circle and a vertical line.

Another critical issue is how good does transfer performance have to be with the novel stimuli to infer solution by a matching concept? The basic idea is that if the animals had learned the original task by adopting a matching concept they ought to transfer rapidly to novel stimuli because a *matching concept* tends to be independent of the stimuli. On the other hand, if the animals had learned the original task using either the *configuration* rule or the *stimulus-response association* rule then performance with the novel stimuli should be poor because both of these processes are dependent on the original stimuli, and indeed it might take the animal as many trials to learn the task with the novel transfer stimuli as it did to learn the task with the training stimuli. Naturally, it is rare that either of these extreme situations prevail, and often we are left with measures of savings from which one must use their best judgment as to what process the animal had employed. For example, if it took an animal 500 trials to learn the original task with circle and vertical line stimuli, and they took 50 trials to learn the task with red and green stimuli, is that sufficiently good performance from which to infer that the original task had been learned using a matching concept? Most would probably say yes. But then what about 100 trials?

There is ample evidence across a wide range of species that animals learn to solve a SMS task by applying a matching concept. Chimpanzees, both adult (Nissen et al., 1948; Robinson, 1955) and infant (Oden et al., 1988), readily transfer to novel stimuli to the point that one could almost talk about near-perfect levels of performance on the first few trials. For example, as a group, the infant chimpanzees in the Oden et al. (1988) study took 816 trials to learn the matching task with the training stimuli to a level of about 85% correct, and continued to score at that level across the first 24 trials with a variety of different novel stimuli. Although not quite to the level of competence of the chimpanzees, monkeys also are capable of showing high levels of transfer with novel stimuli (Mello, 1971; Milner, 1973; D’Amato et al., 1985a).

Outside of non-human primates, studies have focused largely on the abilities of pigeons. Early studies either failed to find evidence of a matching concept (Cumming and Berryman, 1961; Farthing and Opuda, 1974; Holmes, 1979), provided at best weak evidence for a matching concept (Wilson et al., 1985a,b), or the evidence for a matching concept was open to alternative explanations (Zentall and Hogan, 1974, 1978; Urcuioli and Nevin, 1975; Edwards et al., 1983). One such alternative explanation was common coding of stimuli. For example, Zentall and Hogan (1974) trained pigeons with red and green stimuli and then tested with yellow and blue, and the birds showed reasonably good levels of transfer to the “novel” stimuli. Unfortunately, pigeons tend to code yellow and red as similar, and blue and green as similar (Wright and Cummings, 1971), so the transfer seen was nothing more than an instance of stimulus generalization, that is, a violation of the principle or orthogonality. Even a further study (Zentall and Hogan, 1976) in which pigeons trained with a circle and cross geometric forms and then transferred to (clearly novel) red and green stimuli showed high levels of transfer, but failed to recognize that pigeons learn a SMS task with red and green stimuli very quickly (Zentall and Hogan, 1974), thus casting doubt that the rapid transfer to red and green was due to the application of a matching concept.

Early pigeon matching concept studies tended to support the view that, rather than learning a matching concept, the behavior of the pigeons could be best described as learning a series of stimulus-response or configuration associations. The evidence for pigeons forming a matching concept, however, took a big step forward when Wright (1997) showed that the number of responses emitted to the sample stimulus is a critical determinant of whether pigeons will form a matching concept. Different groups of birds were trained to emit either an FR0, FR1, FR10, or FR20 to the sample stimulus, and then tested with novel stimuli under the same response conditions. Wright (1997) found that birds trained with either and FR0 or FR1 failed to transfer to novel stimuli, whereas those trained with FR10 or FR20 showed levels of performance with the novel stimuli similar (or equivalent in the case of the FR20 condition) to their terminal performance with the training stimuli. The number of responses emitted to the sample stimulus was a contextual variable that had been overlooked in many early pigeon studies, where few responses were required to the sample stimulus.

According to Wright (1997), configurational learning is the dominant and preferred learning strategy for pigeons, and in order to display evidence of a matching concept, one must first break the predisposition to process the sample and comparison stimuli as a configuration. Effectively, the larger the FR requirement, the more likely it is that the animal divorces itself from configural learning, and the more likely it will then adopt a matching concept. Take the case of the FR0 condition. The sample and comparison stimuli are presented at the same time, and so it is unlikely that the birds even appreciate that there is a “sample” stimulus that needs to be matched to one of the “comparison” stimuli. And why would they? In effect, the only solution under an FR0 condition is to treat the entire display of “sample” and “comparison” stimuli as a unitary whole, that is, a configuration, and direct your responses accordingly. On the other hand, in the FR20 condition, the sample appears and then only after 20 responses do the comparison stimuli appear. The structure of this task encourages the animals to perceive the sample as something they have to match to the comparison, and as a result, pigeons are more inclined to adopt a matching concept, and transfer to novel stimuli.

A subsequent study by Colombo et al. (2003) uncovered yet another contextual variable that must be adjusted before pigeons will display a matching concept. These authors were surprised when their FR20 pigeons failed to transfer to novel stimuli. They noted, however, that another difference between the Wright (1997) study and their study was that Wright (1997) had initially trained their birds with three stimuli, whereas Colombo et al. (2003) trained theirs with just two. Although training with two versus three stimuli may not seem like an impactful contextual variable, two training stimuli yield four possible sample-comparison configurations, whereas three training stimuli yield 12 possible sample-comparison configurations. Indeed when Colombo et al. (2003) trained another group of birds with three stimuli and an FR20 requirement, they transferred to novel stimuli at a very high level. Thus *number of training stimuli* is also a contextual variable. They reasoned that while it might be possible to learn the right/left responses associated with four configurations, learning the right/left responses associated with 12 configurations might pose difficulty for the animals, and encourage the use of a matching concept to solve the task.

**Figure 1:**
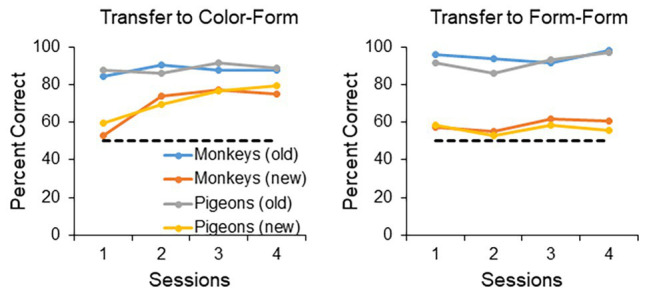
Transfer performance of monkeys and pigeons. The monkey data are based on D’Amato et al. (1985a) and the pigeon data are based on Colombo et al. (2003). The animals were tested over four 48-trial sessions, with half of the trials dedicated to the training (old) stimuli, and half dedicated to the novel (new) stimuli. The **left panel** shows the transfer performance to a novel color and form stimulus (for training the monkeys were trained with two form stimuli and the pigeons trained with three form stimuli). The **right panel** shows the transfer performance to two novel form stimulus (for training the monkeys were trained with a color and a form stimulus, whereas the pigeons were trained with two form stimuli and a color stimulus). When the contextual variables are set appropriately for the pigeons (training with three stimuli and an FR20 on the sample), both monkeys and pigeons transfer readily to a novel color and form stimulus, but not to two novel form stimuli.

In summary, if one designs the experiment properly, one can show levels of transfer in pigeons virtually identical to levels of transfer in monkeys (Colombo et al., 2003). It is true that, in the case of the pigeon, one must impose an FR20 to the sample stimulus and train them with three stimuli, compared to monkeys that show transfer with an FR1 to the sample stimulus and training with just two stimuli. Once these contextual variables are accounted for, however, the performance of pigeons becomes indistinguishable from that of monkeys. This is true not only for the conditions that results in successful transfer, but also the conditions that result in unsuccessful transfer (see Figure 1). Both the D’Amato et al. (1985a) and Colombo et al. (2003) studies employed the same training and testing format, in that the animals were trained with a number of stimuli and then tested over four sessions with novel stimuli as well as the training stimuli. It is clear from Figure 1 that when the contextual variables of FR and number of training stimuli are adjusted, the transfer performance of the birds is indistinguishable from that of the monkeys, both in terms of the successful transfer to a novel color and form stimulus (left panel), as well as unsuccessful transfer to two novel form stimuli (right panel). More on the difference between transfer to color/form and form/form stimuli later.

One final point in the matching concept literature deserves some attention. Premack (1983) has made the claim that animals can be distinguished on the basis of the type of matching procedure that is employed. According to Premack (1983), the procedures discussed in all the above studies are what he calls “successive” matching tasks, where the response of same or different are directed to the physical stimuli themselves (e.g., press the red comparison stimulus if the sample was red). Premack (1983) believes that the ability to solve such “successive” matching tasks is ubiquitous among animals. On the other hand, a “simultaneous” matching task can only be solved, not just by any chimpanzee, but only language-trained chimpanzees. In the “simultaneous” procedure, the discriminanda to which the judgments of same and different must be made are separate from the actual stimuli being judged as same and different. For example, if the animal was presented with stimuli A and B it would have to choose the cue that signifies “different,” say a red rectangle, or if presented with stimuli A and A it would have to choose the cue that signifies “same,” a yellow rectangle (Premack et al., 1978). There seems little doubt that chimpanzees can solve such “simultaneous” tasks (Premack et al., 1978), and despite Premack’s (1983) claim of a language-training prerequisite, so too can non-language-trained monkeys (Sands and Wright, 1980; D’Amato and Colombo, 1989).

Whether pigeons can solve simultaneous matching tasks has, as is often the case for pigeons, taken longer to show. Early positive reports were marred by alternative interpretations, such as the animals potentially learning the fixed order of the left/right responses associated with the “same” and “different” outcomes (Santiago and Wright, 1984), or a failure to fully balance the design thereby allowing the birds to solve the task using item-specific associations (Edwards et al., 1983). Far better transfer performance has been obtained on simultaneous matching tasks when the discriminanda consisted of arrays of multiple same and multiple different stimuli (Santiago and Wright, 1984; Wasserman et al., 1995; Cook et al., 1997). If, in fact, the birds are processing the specific items in the arrays then these studies would provide evidence for pigeons being able to solve simultaneous matching tasks. The criticism with these studies, however, is that the novel “same” and “different” arrays are really not novel. If instead of looking at the individual items that compose an array the animals are processing a global feature, perhaps a measure of the “entropy” of the stimulus array, then the “novel” arrays are really not novel after all (Young et al., 1997). More recently, however, Blaisdell and Cook (2005) have shown that pigeons can perform a simultaneous matching task when only two stimuli are presented at a time, and they transfer to novel stimuli at a level that would suggest evidence of a matching concept.

When Macphail (1985) made his claim that there were no qualitative or quantitative differences among species, he was referring to only vertebrate species. To drive the point home concerning the absence of differences among vertebrates in the ability to form a matching concept, it is worth finishing this section with a matching concept study using invertebrates. Giurfa et al. (2001) showed that honeybees also solve a SMS task using a matching concept. They used a Y-maze with the bees encountering the sample stimulus on the stem of the Y-maze and the comparison stimuli on the arms of the Y-maze. The bees easily learned the task and showed perfect transfer to novel stimuli. So exceptional was the performance of the bees that not only did they transfer to novel visual stimuli but they also transferred the matching concept across modalities, an ability that has never been shown even in non-human primates (see D’Amato et al., 1985a). In summary, when the contextual variables are adjusted for each species, a number of animals display transfer to novel stimuli at a level that would suggest the employment of a matching concept. Whether, in fact, it is necessary to formulate the performance in terms of the cognitive construct of a *matching concept*, as opposed to the operation of associative processes, is an issue to which we will return at the end of this review.

### Symmetry

The second equivalence relation we explore is known as symmetry. When you learn the name of an object, say “door,” from then on, the word “door” brings to mind an image of a door. Likewise an image of a door brings to mind the word “door.” This is an example of *symmetry*, a bidirectional association between two stimuli. Symmetry in the context of the animal literature is usually trained using a version of the matching-to-sample task called the symbolic or conditional matching-to-sample, in which different sample stimuli are mapped onto different comparison stimuli. The aim of the task is therefore not to match, in terms of sameness, a comparison stimulus to a sample stimulus, but to choose the comparison stimulus that is associated with the sample stimulus. For example, if A1 and A2 are the sample stimuli, and B1 and B2 are the comparison stimuli, then when A1 appears as the sample the correct choice is B1, whereas when A2 appears as the sample the correct choice is B2. To test for symmetry, B1 and B2 now become the sample stimuli, and A1 and A2 become the comparison stimuli. If the learned relationships, A1→B1 and A2→B2 are symmetrical, then when presented with B1 or B2 as the sample stimuli the subject should chose A1 and A2, respectively. Although animals readily learn symbolic matching-to-sample tasks, demonstrating symmetry in a number of species has proven difficult.

It is worth mentioning at this point that the term symmetry typically implies that the backward association is learned to the same degree as the forward association. By this harsh definition, it would appear that there is little or no evidence for such symmetry in nonhuman animals. As in most cases, in the nonhuman animal literature we accept a significant backward association (albeit less pronounced than the forward one) as evidence of symmetry. With this in mind, Tomonaga et al. (1991) trained three chimpanzees to match one of two sample colors to one of two comparison shapes to a criterion of at least 80%, then overtrained the animals for hundreds of trials, and then tested for symmetry over 12 trials. Keep in mind that testing for the emergence of an ability over a mere 12 trials is a tall order, as animals are often impaired by any change in testing conditions. Nevertheless, one of three trained chimpanzees performed above chance on the symmetry test, providing evidence that chimpanzees are capable of forming symmetrical relations. The evidence for symmetry in chimpanzees, however, is by no means uniformly positive. Yamamoto and Asano (1995), for example, found their one chimpanzee displayed no evidence for symmetry after training with one stimulus set, but after specific training and testing with six stimulus sets, a procedure called exemplar training, symmetry did emerge.

Demonstrating symmetry in monkeys has also been met with great difficulty. Sidman et al. (1982) failed to show any evidence of symmetry in monkeys trained with geometric (vertical and horizontal line) samples and color comparison stimuli. McIntire et al. (1987) purported to show evidence of symmetry in macaque monkeys; however, their conclusions were met with considerable criticisms on the basis that the tested-for relations were already trained (see Hayes, 1989). Surprisingly, the study by D’Amato et al. (1985b), one that is commonly cited as a negative finding (Hayes, 1989; Sidman, 1994; Lionello-DeNolf and Urcuioli, 2002; Frank and Wasserman, 2005), provides some favorable evidence for symmetry in monkeys. D’Amato et al. (1985b) argued that the use of vertical and horizontal line comparison stimuli in the Sidman et al. (1982) study could have been the contextual variable that put the monkeys at a disadvantage. Employing far more discriminable stimuli as sample and comparisons, and also assessing performance over the first 12 trials, D’Amato et al. (1985b) showed evidence for significant backward associations in two of the six monkeys tested.

Numerous studies have explored the extent to which pigeons display symmetry, and positive findings have been difficult to obtain. Early studies either failed to find any evidence for even backward associations (Lipkens et al., 1988), were criticized for alternative interpretations when they did (Vaughan, 1988; Hayes, 1989) or much like for chimpanzees and monkeys, found at best only weak evidence for backward associations (Hogan and Zentall, 1977; Richards, 1988). Interestingly in the Hogan and Zentall (1977) study, some of the positive evidence for symmetry was seen early in the test for symmetry but then dissipated, an outcome also observed by D’Amato et al. (1985b) with monkeys. Given the context of this article it is perhaps fitting to include one possibility raised by Hogan and Zentall (1977, p. 14) as to why the pigeons fare poorly on symmetry tasks: “it is also possible that the development of backward associations depends upon the species-specific functional value of such associations (i.e., humans may need to be able to develop backward associations whereas pigeons may not).”

Lionello-DeNolf and Urcuioli (2002) also failed to find evidence for symmetry in pigeons, but their study is worth mentioning because it represents one of the earliest attempts to address possible contextual variables that may be preventing pigeons (and possibly other animals) from displaying symmetry. Drawing from McIlvane et al.’s (2000) notion of *stimulus response topography* that pigeons may process aspects of a stimulus that interfere with the aspects of interest in tests of symmetry, Lionello-DeNolf and Urcuioli (2002) reasoned that during the symmetry test not only do the sample and comparison exchange roles but they also exchange positions. Thus, pigeons seem to code not only the features of the stimulus but also the positions of the stimuli as part of the *stimulus response topography*. Take the situation in matching tasks where the sample stimulus typically appears in a central position and the comparison stimuli appear to either side of the sample position. For the test of symmetry, the comparison stimuli now appear in the central position. To a human it might be irrelevant that the comparison stimulus now appears in a position that it has never appeared in before, but to a nonhuman, position may be part of the *stimulus response topography*, and hence, nonhuman animals may fail the symmetry test because it is unclear how they should behave when stimuli appear in positions that they have never appeared in before. Lionello-DeNolf and Urcuioli (2002) therefore trained their animals so that the sample and comparison stimuli could appear in any of a number of positions, thus effectively training “position” out as a component of the *stimulus response topography*. Despite this training the pigeons still failed to show any evidence for symmetry, a finding that, marginal as the evidence for symmetry is in non-human primates, further seems to distance pigeons from nonhumans in their ability to form symmetrical relations.

Frank and Wasserman (2005) noted, however, that in addition to the stimuli being associated with their spatial location, they are also associated with their temporal location. In other words, if the relation A1→B1 is trained and then the relation B1→A1 is tested, item B has never appeared first. Similar to the case for position mentioned earlier, if item B now appears first, we as humans may quickly assume that because it appears first it must be serving in the role of a sample stimulus, but again there is no reason why other animals should make that assumption. To account for the potentially controlling influence of the contextual variable of temporal location, Frank and Wasserman (2005) used a successive go/no-go matching tasks, where the sample and comparison stimuli appear successively in the same position, and the subject required to make a go response to the second stimulus if it is paired with the first (e.g., A1→B1), and a no-go response (i.e., withhold responding) to the second stimulus if it is not paired with the first (e.g., A1→B2). To control for the potentially disruptive effects of the contextual variable of temporal order and the fact that, for example, stimulus B had never appeared first, the pigeons were trained not only with symbolic relations (A1→B1, A2→B2) but also with identity relations (A→A and B→B), thus training the animals that both stimuli A and B can occur in any temporal position. With these contextual variables in mind, the pigeons displayed robust symmetry. Frank and Wasserman (2005, p. 157) concluded that “symmetry can be obtained with nonhuman animals under proper conditions of training and testing.” Interestingly, the one successful chimpanzee in the Tomonaga et al. (1991) study was also trained with both symbolic and identity relations.

In summary, Frank and Wasserman’s (2005) elegant study shows that once contextual variables are taken into account, pigeons can display symmetry, and do so to a level not that dissimilar to chimpanzees. Furthermore, there is little if any evidence that would distinguish the performance of non-human primates and birds with respect to the formation of symmetry. Much like the matching concept literature, researchers are now investigating whether symmetry can be demonstrated by invertebrates. Given a recent attempt by Moreno et al. (2012) with honeybees, it seems only a matter of time before an invertebrate species can be shown to display symmetry.

### Transitivity

The third equivalence relation we explore is known as transitivity. There is little need to appeal to the notion of contextual variables because most species have been shown capable of solving transitivity tasks. Rather, we include a brief mention of this topic to complete our discussion of equivalence relations, and more importantly to highlight another issue we wish to briefly address in this review, namely the desire to interpret the behavior of nonhuman animals in overtly cognitively-rich terms.

Transitivity is an operation whereby given the information that A is smarter than B, and B is smarter than C, one makes the logical conclusion that A is smarter than C, even though no direct information about the relationship between A and C was ever given. According to Piaget (1928), the ability to solve such a three-term transitive inference task does not develop until approximately 7 years of age, a conclusion that was challenged by Bryant and Trabasso (1971), who demonstrated robust transitive inference abilities in 4-, 5-, and 6-year olds. Although the main purpose of this review is to compare nonhuman animals, the procedure used by Bryant and Trabasso (1971) is worth mentioning because very similar training procedures have been used to explore transitivity in nonhuman animals. In their study, children were trained to discriminate between colored rods of different lengths. The rods were presented in pairs, and training consisted of repeated exposures to four training pairs, A+B−, B+C−, C+D−, and D+E−, with the letters representing the different lengths of rods (e.g., A was the longest and E the shortest) and the “+” and “−” indicating the correct and incorrect stimulus, respectively, to select. For example, when presented with pair CD and prompted with the question “*which rod is longer?*”, the subject should select item C.

Of course, in learning a five-item transitive series, A is always correct, E is always incorrect, and B, C, and D are both correct and incorrect depending on the pair in which they appear. With a five-item series there are 10 possible pairs with which to test the subject (AB, AC, AD, AE, BC, BD, BE, CD, CE, and DE). Of these, we expect the subject to perform well with any pair that contains item A (AB, AC, AD, and AE) because in training item A was always correct. We also expect the subjects to perform well with any pair that contains item E (AE, BE, CE, and DE) because in training item E was always incorrect and hence should always be avoided in favor of the other stimulus. Finally, we clearly expect them to perform well with a pair that was one of the training pairs (AB, BC, CD, and DE), leaving as the critical test for transitivity pair BD. Bryant and Trabasso (1971) found that 4-, 5-, and 6-year olds performed at high levels on pair BD.

Studies using nonhuman animal subjects tend to follow the same general procedure adopted by Bryant and Trabasso (1971) of initially training the animals on the four premise pairs AB, BC, CD, and DE, and then testing them on the critical BD pair. Using this procedure chimpanzees (Gillan, 1981), monkeys (McGonigle and Chalmers, 1977), rats (Davis, 1992), and pigeons (von Fersen et al., 1991; Paz-y-Miño C et al., 2004) have all been found to perform at high levels on the critical BD test pair, and indeed achieve levels of performance not too different from that reported by Bryant and Trabasso (1971) for young children. To be sure there have been failures by pigeons to show transitivity (D’Amato et al., 1985b), but there have also been failures by primates to display transitivity (Sidman et al., 1982). Despite the occasional failure, there is no need to appeal to contextual variables, because in general pigeons solved transitivity tasks as well as other animals.

A key feature surrounding many of these studies is the extent to which the high level of performance on the BD test pair reflects a cognitive/logical operation or a behavioral/associative operation. In the cognitive/logical camp is the view that while learning how to respond to each of the five premise pairs (e.g., A+B−, B+C−, C+D−, and D+E−) animals form a hierarchical linear mental representation of how the five stimuli are related to one another (e.g., A > B > C > D > E), and use that representation to guide them as to how to respond to the critical BD test pair. In the behavioral/associative camp, no linear mental representation of the five items is formed. Rather, solution of the critical BD pair is based on conducting an associative computation based on reward values assigned to each of the items (Value Transfer Theory), or by relying on previously learned premise pairs to solve the BD problem (Binary Sampling Model). According to the Value Transfer Theory (von Fersen et al., 1990, 1991), different strengths are assigned to each of the five stimuli as a function of which pairs they have appeared in during training and whether they were associated with the always rewarded stimulus A or never-rewarded stimulus E. As a result of such associations, item B is ranked higher than item D, and so the animal will choose B when presented with pair BD. Indeed, the resulting rankings can be used to very accurately predict which stimulus an animal will select when any two stimuli are paired.

**Figure 2:**
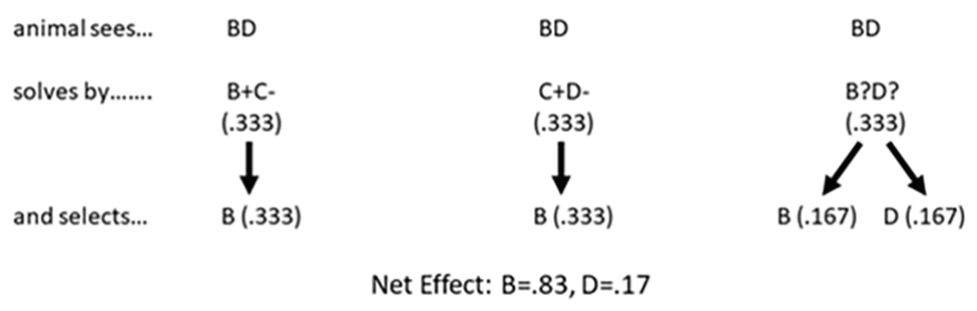
The Binary Sampling Model. According to McGonigle and Chalmers (1977), an animal attempts to solve the BD pair as if it were either pair BC, CD, or BD. B+C− is a training pair and so it will select item B. C+D− is also a training pair where the animal is taught to select C. Unfortunately, because the animal has been presented with pair BD, there is no item C being displayed and hence no item C to select. But if the animal were trying to solve the BD pair as if it was item C+D− being displayed, it also learned to avoid D, which is what the animal does, and again selects item B. Finally, if the animal attempts to solve the BD pair as if it were BD, it has received no training with these two stimuli presented together, and randomly chooses between them. The net effect is the animal will select B 83% of the time and D 17% of the time, which happens to be very close the performance levels many animals achieved with the BD test.

The Binary Sampling Model (McGonigle and Chalmers, 1977) is also a simple yet effective noncognitive account of why an animal selects item B during the critical BD test. According to the model (see Figure 2), upon seeing pair BD the animal attempts to solve the task as if it were either pair BC, CD, or BD. A test session typically consists of numerous presentations of pair BD, and according to the model, there is a 1 in 3 chance that either of the three pairs is selected on any given trial. Given that each pair is selected 33% of the time, we can think of each pair as having 0.33 units to contribute to the solution of the BD problem. If the animal attempts to solve the BD pair as if it were pair BC, it will select B because B+C− is one of the training pairs where the animal is taught to select B. Item B therefore accumulates 0.33 units. If the animal attempts to solve the BD pair as if it were pair CD it will select C because C+D− is one of the training pairs where the animal is taught to select C. But keep in mind that there is no item C to select because, remember, the animal is presented with pair BD not CD. However, if the animal was attempting to solve pair BD as if it were pair CD the animal is also trained on that pair to avoid item D, and so the animal avoids item D in pair BD and selects item B. Item B again gets all 0.33 units, bringing its current tally to 0.66 units. Finally, the animal may attempt to solve pair BD as if it was pair BD. Unfortunately, pair BD is not a training pair and so no associations have been established between items B and D, and the animal will randomly select B half the time and D half the time, and the 0.33 available units gets split between the two items. The final tally is that item B gets 0.83 units and item D gets 0.16 units, which when expressed in terms of percent correct is remarkably close to the performance of animals with the BD test pair across a wide range of studies.

Both the Value Transfer Theory and Binary Sampling Model, as well as other noncognitive accounts of transitivity (see Delius and Siemann, 1998), very nicely account for the high levels of BD test pair performance without the need to appeal to cognitive accounts such as hierarchical mental representations. Of course, there have been challenges to these simpler accounts of transitive inference (Steirn et al., 1995; Lazareva and Wasserman, 2012) but it is hard to overlook the power of associative strength *via* reinforcement history (Siemann et al., 1996). It is difficult to do justice in this review to the complex transitive inference literature, but whether one believes in cognitive/logical accounts or behavioral/associative accounts, one thing is certain, there is no support for the view that monkeys perform such tasks any differently to pigeons. The recent demonstration of this ability in invertebrates means this question can now be extended beyond vertebrates (Tibbetts et al., 2019). Perhaps more importantly, the fact that a species with just 0.001% of the neurons in a human brain (Azevedo et al., 2009; Menzel, 2012) can pass the task should call into question the cognitively-rich terms with which researchers describe transitive inference.

### Serial-Order Behavior

Conceptually related to studies of transitivity are studies that explore the serial-order abilities of animals. The serial-order task, also known as the simultaneous chaining procedure, has provided a wealth of information on the structure of the representations believed to underlie transitive judgments. The task is straightforward, and like the transitivity procedure, often uses five stimuli. Rather than presenting the five stimuli as four training premise pairs, however, in the serial-order task the animals are trained to respond to five simultaneously presented stimuli in a specific order, namely, A→B→C→D→E. Both monkeys and pigeons can learn to perform the five-item serial-order tasks to the same high levels (D’Amato and Colombo, 1988; Terrace 1993; Scarf and Colombo, 2010). To determine what the animals have learned, much like in the transitivity test, subjects are given a pairwise test consisting of all 10 possible pairs of stimuli that can be generated from the five-item list (AB, AC, AD, AE, BC, BD, BE, CD, CE, and DE). A correct response on the pairwise test requires that the animals respond to the two displayed items in a manner consistent with their order in the five-item series. When presented with pair BC, for example, to obtain a reward the animal must first respond to item B and then to item C.

**Figure 3:**
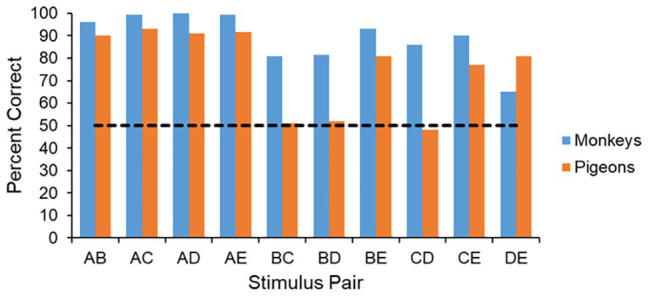
Performance across the 10 pairs during the pairwise test. The monkeys perform well on all pairs, whereas the pigeons only perform well on pairs that have either an item A or an item E, and perform at chance on the internal pairs that are missing these items.

The pairwise test has provided considerable insight into the processes that different animals use in learning the original five-item serial-order task. In fact, until recently, the performance on the pairwise tests, as well as latency measures that can generated from the correct responses, seemed to provide some of the best evidence that monkeys and pigeons process serial-order information in fundamentally different ways (Terrace, 1993; Scarf and Colombo, 2008). For example, in terms of performance across the 10 pairs, monkeys perform at very high levels on all test pairs, whereas pigeons perform at high levels only on pairs that contain either item A or item E. Importantly, pigeons perform at chance levels on the internal pairs BC, BD, and CD (see Figure 3). Such an outcome is consistent with the view that in the course of learning a serial-order task monkeys form a mental representation of the list and used that representation to guide their behavior (D’Amato and Colombo, 1988). Pigeons, on the other hand, seem unable to form such a representation, and rather learn a simple set of behavioral rules such as “(1) Respond first to item A. (2) Respond last to item D. (3) Respond to any other item by default” (Terrace, 1993, p. 164).

**Figure 4:**
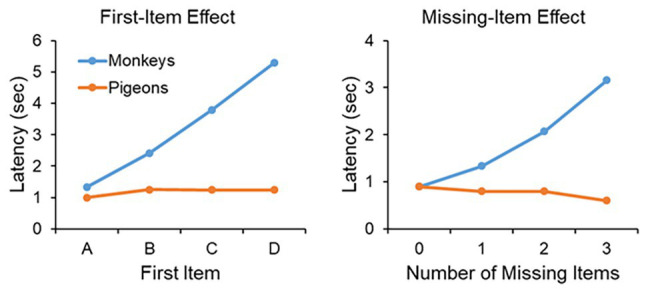
**Left panel**: The *first-item effect*. The latency to respond to the first item of a pair for monkeys and pigeons as a function of whether the first item was A (averaged across pairs AB, AC, AD, and AE), B (averaged across pairs BC, BD, and BE), C (averaged across pairs CD and CE), or D (based on pair DE only). Monkeys show a linear increase across first item whereas pigeons do not. The data on based on correct trials. **Right panel**: The *missing-item effect*. The latency to respond to the second item of a pair as a function of whether the second item was separated from the first by 0 missing items (averaged across pairs AB, BC, CD, and DE), 1 missing item (averaged across pairs AC, BD, and CE), 2 missing items (averaged across pairs AD and BE), or 3 missing items (based on pair AE only). Monkeys show a linear increase across the number of missing items whereas pigeons do not. The data on based on correct trials.

Further evidence that monkeys form a mental representation of the series comes from two types of analysis of the latency data to respond to the first and second items of the displayed pair. In the case of the *first-item effect*, the latency to the first item of the pair is averaged across all pairs that share the same first item. In other words, the latency to item A is averaged across pairs AB, AC, AD, and AE, the latency to item B averaged across pairs BC, BD, and BE, the latency to item C averaged across pairs CD and CE, and the latency to item D is based on the only pair that has item D as a first item, pair DE (see Figure 4, left panel). Monkeys clearly display a *first-item effect*, in that the latency to respond to the first item of a pair is longer the further along the list that the first item lies. For example, the latency to respond to item C in pair CD takes longer than the latency to respond to item B in pair BD. Such a latency function suggests that the monkeys are accessing the list at item A and progressing through the list in a linear fashion trying to match the item in memory to a displayed item. In contrast to monkeys, pigeons show a flat *first-item effect*.

Monkeys also display what is known as a *missing-item effect* (Figure 4, right panel). The *missing-item effect* refers to the latency to respond to the second item of a pair as a function of the distance from the first item to the second item. In pairs AB, BC, CD, and DE, there are no missing items in that the second stimulus of a pair occurs directly after the first stimulus. Pairs AC, BD, and CE have one missing item, pairs AD and BE have two missing items, and pair AE has three missing items. Monkeys display a very clear *missing-item effect* in that the latency to respond to the second item of a pair is a function of the number of missing items between the first and second item. For example, monkeys are faster to respond to item D in pair CD than item D in pair BD. The reason is because in pair CD there are no missing items to access, whereas in pair BD the monkey must access one missing item, item C. In contrast to the monkeys, pigeons do not display a *missing-item effect*.

The performance across the 10 pairs, as well as the presence of a *first-item effect* and a *missing-item effect*, supports the view that in the course of learning a serial-order task monkeys form a linear mental representation of the items and use that representation to guide their behavior, for example, during the pairwise test. In contrast, the absence of these effects in pigeons suggests that they solve the serial-order task in a fundamentally different way to monkeys. These views fit well with the notion that the success of the monkeys may very well be related to their ability to respond appropriately to dominance hierarchies (Cheney et al., 1986), something that is not necessary for the pigeon, whose social structure has a far less hierarchical organization (Masure and Allee, 1934).

Is it really the case, however, that pigeons have no knowledge of the ordering of the stimuli in a serial-order task, or has revealing that ability been masked by some contextual variable? Recall that the pairwise test occurs once the animals have reached a certain level of proficiency on the five-item serial-order task, and consists of presenting the subjects with all 10 pairs of stimuli that can be generated from the five items (AB, AC, AD, AE, BC, BD, BE, CD, CE, and DE). Furthermore, each of the 10 pairs is shown a number of times within a session (typically four times within a 40-trial session). We wondered whether the structure of the pairwise test, and the surprise at being shifted from a five-item task to a pairwise test with all 10 pairs intermixed within a session, was perhaps causing the pigeons difficulty. Was the dramatic change in context the contextual variable that accounted for the pigeons’ poor performance on the pairwise test? We explored this possibility across two experiments (Scarf and Colombo, 2010).

**Figure 5:**
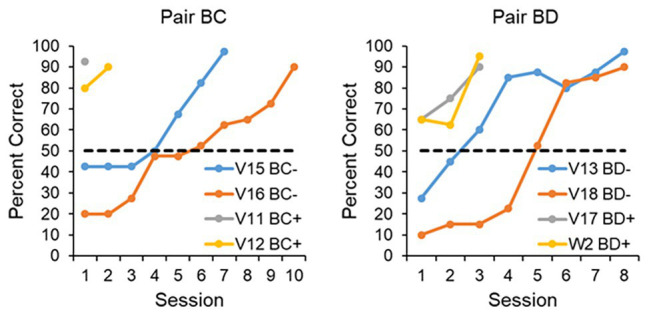
Performance on pair BC after training on a four-item list (**left panel**) and BD after training on a five-item list (**right panel**). Two animals each were trained on the BC+, BC−, BD+, and BD− conditions, where the “+” indicated that a reward could be obtained by responding to the items in the order in which they appeared in the original sequence (B→C or B→D), and the “−” indicated that a reward could be obtained by responding to the items in the order opposite to what they appeared in the original sequence (C→B or D→B). If the birds learned nothing about the order of the internal items, then when presented with these pairs they ought to take as long to learn the positive pair condition as the negative pair condition. Rather, it is clear that the birds tested with the positive pairs fared far better than those tested with the negative pairs.

In one experiment, we trained four pigeons on a four-item serial-order task and another four pigeons on a five-item serial-order task. Instead of then delivering a pairwise test of six pairs (the number that can be generated from a four-item list) for the birds trained on the four-item task, or 10 pairs for the birds trained on the five-item task, we attempted to mitigate the effects of the context change by presenting the four-item-trained birds with just pair BC (the critical internal pair after training on the four-item list) or the five-item-trained birds with just pair BD (a critical internal pair after training on a five-item list). The BC or BD pairs were presented 40 times per session. We reasoned that if the pigeons learned nothing about the order of items B and C, or the order of items B and D, then those tested on the positive pair condition (BC+ or BD+) and rewarded for pressing B→C or B→D should fare no better than those tested on the negative pair condition (BC− and BD−) and rewarded from responding to the items in the opposite direction, that is C→B or D→B. The results are shown in Figure 5. Clearly animals trained on the positive pairs acquired the task significantly faster than those trained on the negative pairs, suggesting that if the conditions are set up properly, pigeons display evidence that they understand the order of the internal items on four-item and five-item serial-order tasks.

**Figure 6:**
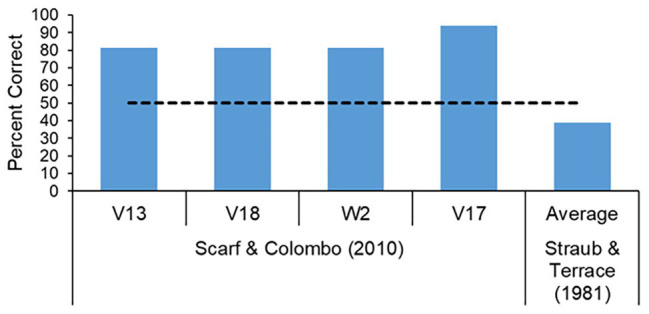
Performance on pair BC when delivered as probes embedded against a baseline four-item serial-order task. All four pigeons performed at high levels after exposure to only 16 BC probes trials. Also shown is the BC performance of Straub and Terrace (1981) pigeons where the BC trials were delivered in a standard pairwise tests fashion along with all the other pairs in a session.

When tested with just one pair, the birds were able to indicate that they did understand that item B comes before item C, or item B comes before item D, and thus provide us with evidence that they did understand, at least at some rudimentary level, the organization of the internal items in a series. That said, the positive pair birds did experience far more trials (40–80 for the BC+ birds, 120 for the BD+ birds) on their respective pairs than that typically experience by the monkeys on those same pairs during a regular pairwise test (usually around 8–12 trials). We wondered, then, if pigeons could ever display high levels of performance on a critical pair, as did the monkeys, following exposure to a limited number of trials. To test this notion we again modified the pairwise testing procedure. For the second experiment, pigeons were trained on a four-item serial-order task and presented with the critical BC pair as a probe of four trials embedded against a baseline of 36 trials dedicated to the standard (A→B→C→D) four-item serial-order task. The test was run for four sessions giving a total number of 16 BC trials, a number very similar to that experienced by the monkeys. The results are shown in Figure 6. All four birds performed at very high levels across the 16 BC probe trials. For comparison, also shown in the figure is the performance of pigeons on the BC pair when it was delivered in a standard pairwise test format in which the six pairs that can be generated following training with a four-item list (AB, AC, AD, BC, BD, and CD) are presented intermixed within a session (Straub and Terrace, 1981) with no baseline A→B→C→D trials. Clearly, pigeons can perform well on a critical pair after limited exposure to that pair, but only when the context of the overall test is not dramatically changed from the training situation.

We have shown that by mitigating the effects of a dramatic change in context, pigeons can perform well on a critical internal test pair, thus supporting the view that they do understand the order of the internal items in a list. It would seem that for the pigeons, for whatever reason, displaying all the pairs at once as in a standard pairwise test is a contextual variable that prevents them from displaying their understanding of the organization of the items of a four-item and five-item lists.

### Episodic Memory and Theory of Mind

There are many tasks that have been used to probe the abilities of nonhuman animals, for which there are not only no differences in performance across species, but also for which some of the most compelling evidence for a particular ability actually comes from birds, rather monkeys or chimpanzees. Such experiments speak very clearly to the *Null Hypothesis*. A case in point is episodic memory. Episodic memory refers to the recollections of personal experiences of one’s life. Tulving (1972) originally envisioned episodic memory as consisting of memory for *what* the event was, *where* the event occurred, and *when* in one’s life the event happened, colloquially referred to as WWW memory. Later, Tulving (1985) refined his definition to include the concept of autonoetic consciousness (autonoesis), the phenomenological experience that the memory one retrieves is indeed something that has happened to you in the past. If episodic memory is defined as requiring autonoesis, which can only be accessed by a verbal report, then it is unlikely that any nonhuman animal can satisfy the criterion for possessing episodic memory. However, if we revert to Tulving’s (1972) original definition of episodic memory as memory for *what*, *when*, and *where*, then there is accumulating evidence that a variety of animals possess episodic memory, or at least what some have cautiously referred to as *episodic-like* memory.

The Clayton and Dickinson (1998) study still ranks as the most compelling evidence to date that nonhumans, in their case scrub jays, can use *what*, *where*, and *when* information to guide their behavior. Since the publication of the Clayton and Dickinson (1998) study, there have been many other attempts at showing WWW-memory in a number of species such as rats (Bird et al., 2003; Babb and Crystal, 2005; Ergorul and Eichenbaum, 2007), pigeons (Skov-Rackette et al., 2006), monkeys (Hampton et al., 2005), and apes (Schwartz et al., 2002, 2004, 2005; Mulcahy and Call, 2006). In many cases, these experiments have alternative explanations that do not necessitate the attribution of episodic memory (see Colombo and Hayne, 2010). In others, the evidence can be tantalizingly close to that of the Clayton and Dickinson (1998) study with jays (Mulcahy and Call, 2006), but always seems to fall just short of the performance of the jays, although some of the more recent work by Crystal and his colleagues raises rats’ abilities on par with those of the jays (for a review see Crystal, 2011). The same is true for the ability to plan for a future need, which was very elegantly shown in jays (Raby et al., 2007) and then chimpanzees (Inoue and Matsuzawa, 2007), and more recently in rats (Crystal, 2013). It is also important to bear in mind that even the Clayton and Dickinson (1998) study is not without its critics who oppose the view that the jays are displaying episodic memory (Suddendorf and Busby, 2003; Suddendorf and Corballis, 2007). Nevertheless, with proper experimental designs in place, it is simply a matter of time before all animals show high levels of proficiency on WWW tasks.

Studies exploring the capacity of animals to display Theory of Mind (ToM) is another example where birds display remarkable abilities. Premack and Woodruff (1978) posed the question: “*Does a chimpanzee have a theory of mind?*” On the basis of the ability of chimpanzees to select the proper picture depicting a solution to a previously seen 30-s video clip of a person facing a dilemma, the authors concluded that chimpanzees do have a ToM. Similarly, Povinelli and colleagues compared two forms of mental state attribution, role reversal and the concept of a knower versus a guesser (Povinelli et al., 1990; Povinelli, 1993). In the case of the role reversal experiment, the chimpanzees were able to appreciate not only their role in securing food, but also that of the human they were paired with, so that if switched to the other’s role, they still succeeded in obtaining food. Likewise, in the knower-guesser experiment, the chimpanzees provided evidence that they understood that the person who remained in the room (the knower) had knowledge of the whereabouts of the hidden food, whereas the person that left the room (the guesser) did not, so that when given the choice they chose the location indicated by the knower rather than the guesser.

Although neither the video-clip, role-reversal, nor knower-guesser experiments have been conducted with birds, Emery and Clayton (2001) did examine the effects of experience and social context on the ability of scrub jays to cache food. Jays were given the opportunity to cache food either in the presence of an observer jay or in private. The authors found that jays were far more likely to recache their food if they had previously cached while being observed, suggesting that they understood the intentions of the observing jay. Indeed, only those jays that themselves had experienced pilfering caches displayed such an ability, whereas naïve jays did not recache any more in the observed condition than the in-private condition. These results support the age-old adage that “It takes a thief to know a thief,” and highlights the remarkable ability of these birds with respect to mental state attribution.

To be sure there are critics of all these studies, indeed Povinelli (1994) has since conceded the chimpanzees may have learnt to respond to a behavioral cue rather than infer each of the experimenters’ knowledge state, a far simpler take on ToM than mental state attribution. In a critique of the ToM literature Heyes (1998, p. 101) evaluated the empirical evidence that chimpanzees possess a ToM and concluded that “in every case where non-human primate behavior has been interpreted as a sign of ToM, it could instead have occurred by chance or as a product of nonmentalistic processes such as associative learning or inference based on nonmental categories.” And similarly, the findings of Emery and Clayton (2001) can also be attributed to simple learning processes and associations. Although we subscribe to these simpler interpretations, the main point we wish to make now, however, is that there is no evidence to suggest that a particular capacity such as episodic memory, or ToM (or any of the previous abilities we have discussed) is present in one species and not another.

## Macphail Revisited

Our review is not exhaustive in the sense that we have not examined every task on which species have been compared. For example, how different species perform on habituation, classical conditioning, and instrumental conditioning tasks, what Macphail labeled as “simple” tasks, have been extensively reviewed by Macphail (1982, 1985, 1987), and it was not our intention to go over those again, mainly because there is probably little disagreement that vertebrates perform similarly on such “simple” tasks. Rather, our goal was to evaluate Macphail’s (1985) *Null Hypothesis* in light of the recent explosion of interest in the mental abilities of nonhuman animals, and the tasks that have been used to infer these abilities. These tasks are those referred to by Macphail as “complex” tasks, and Macphail recognized that disagreement over his *Null Hypothesis* would focus on these “complex” tasks.

We have reviewed a large number of such “complex” tasks such as reflexivity (matching concept), symmetry, and serial-order behavior, and have shown that differences in performance between species can be traced to a contextual variable, be it the FR requirement to the sample stimulus or the number of training stimuli in the case of reflexivity, aspects of the stimulus response topography in the case of symmetry, or the testing situation in the case of serial-order behavior. For other tasks, such as transitivity, episodic memory, and ToM, the performance of birds rivals, and at times exceeds that of non-human primates. Our review of the literature indicates that there is very little difference in the performance on these “complex” tasks across a range of vertebrate species. On the basis of the above review, and notwithstanding the potential pitfalls inherent in all such comparisons, we agree with Macphail (1985, p. 39) when he stated that “there is currently no phenomenon of learning demonstrable in one (non-human) vertebrate species that has not been found in all other vertebrates in which it has been sought systematically.”

### Qualitative Differences Versus Quantitative Differences

*By a qualitative difference between species is meant the possession by one species of a mechanism that is absent in another…. A quantitative difference between two species would mean that one species used a mechanism or mechanisms common to both species more efficiently than the other,” (Macphail, 1985, p. 38).*

We do not mean to imply that there are no instances of a particular task in which the performance of one species exceeds that of another. Indeed, there are many such cases. It is hard to escape the fact, therefore, that species do differ quantitatively. The ease with which chimpanzees and monkeys can learn tasks is all too apparent, and although speed of learning is not the best proxy for cognitive abilities, it does speak to some difference in processing capacity, even once issues such as contextual variables are account for. And the mere fact that a pigeon needs a testing situation set up in a specific way, whereas a monkey may not, further speaks to a quantitative difference at the phenomenological level, and possibly also at the process level (see further discussion below). These quantitative differences also surely extend to the range of transfer situations with a more restricted range in pigeons than that seen in monkeys, and indeed a more restricted range in monkeys than that seen in chimpanzees or humans (Weinstein, 1941). And after all, it is the ability to transfer to novel situations, which is really the hallmark of what we call intelligence, and in this respect the abilities of humans exceeds that of monkeys, just as the abilities of monkeys more than likely exceeds that of pigeons. Indeed, we would argue that the main difference in “intelligence” among animals lies in the degree to which one must account for contextual variables, which in turn reflects the level of flexibility of an animal’s behavioral repertoire. Surely, the extra cortical tissue of a primate brain, even once one accounts for body size, is what allows it to express behaviors in less restricted manners, and surely that is what lies at the heart of “intelligence.”

### Associative Processes or Cognitive Processes?

An important point to bear in mind when comparing the performance of species on a particular task is that similar looking graphs do not imply similar underlying processes. Just because a pigeon shows levels of transfer on a matching task similar to that of monkeys, or performs similarly on tests of transitivity, does not mean that it is invoking the same processes to solve the task as a monkey. A similar point was trenchantly put forth by Gallup in his reply to Epstein et al.’s (1981) demonstration of self recognition abilities in pigeons when he stated that “Simply because you can mimic the behavior of one species by reinforcing a series of successive approximations to what looks like the same routine in another, it does not follow that the behavior of the former species necessarily arose in the same way” (Gallup, 1985, p. 633). Although a fair criticism, the simple fact is that there is virtually no evidence to suggest that pigeons are solving complex tasks differently from monkeys, or monkeys differently from chimpanzees, once, of course, contextual variables are taken into consideration. The fact that pigeons, monkeys, and chimpanzees are solving tasks similarly is supported not only by the success-testing metric, but also more importantly by the signature-testing metric, which explores the various signatures of performance on a task (Taylor, 2014; Scarf and Colombo, 2020).

The important question for comparative cognition is not whether an animal can solve a task or not, but rather *how do they solve tasks*? We invoke constructs, such as a *matching concept*, *symmetry*, *transitivity*, and *orthographic processing* as if these constructs are explanations of behavior. They are not, they are just labels for a behavior. Epstein et al. (1981, p. 696) put it beautifully when they said that “such constructs impede the search for the controlling variables of the behavior they are said to procedure.” The temptation to richly interpret an animal’s behavior is pervasive (Haith, 1998; Shettleworth, 2010). Speaking for our own research, we may argue that pigeons have a matching concept (Colombo et al., 2003), abstract numerical abilities (Scarf et al., 2011), and orthographic processing (Scarf et al., 2016), but we do not believe that pigeons (or monkeys) succeed on such tasks because they have advanced cognitive skills. Rather we use these constructs, much in the same way that Skinner, Epstein, and their colleagues used them in the Columban simulation studies (self-awareness: Epstein et al., 1981; symbolic communication: Epstein et al., 1980; insight: Epstein et al., 1984), to mimic the constructs that have been used with primates, for whom we feel much more comfortable adopting such labels.

If not “cognitive” processes, then what processes underlies these impressive abilities? We surely underestimate the power of simpler (but not simple) accounts such as associative learning or reinforcement-learning processes (Dickinson, 2012; Hanus, 2016; Haselgrove, 2016). We doubt that our pigeons (or the monkeys) are truly engaging in “orthographic processing” and breaking down each four-letter word they see into its constituent pairs, and evaluating the frequency with which each pair is likely to occur in words or nonwords (Grainger et al., 2012). Rather, we agree with Vokey and Jamieson (2015, see also Linke et al., 2017) that the monkeys and the birds are likely mapping novel words onto prototypic “word” and “nonword” templates, an impressive and certainly not a simple ability to be sure, but one that differs from an “orthographic” account. Similarly, we might invoke “mental representations” as processes governing the behavior of pigeons and monkeys on a transitivity task, but simpler accounts such as Value Transfer Theory and Binary Sampling Model go a long way to explain the behavior. True these simpler accounts may not explain every nuance of a behavior that has been observed (and they should), but how much of that might reflect our lack of understanding of these simpler accounts, as opposed to a shortcoming of these simpler accounts?

The issue we touch on above is a critical issue for comparative cognition, and it is impossible to do it justice as a side note of a few paragraphs. We agree with Allen (2014, p. 76) that there is too much “trophy hunting,” and that those theories that are available are not formalized to a sufficient degree to truly untangle the difference between associative and cognitive models of behavior. But models are critically important if we are to advance the field, especially process-based models (Luce, 1995; Buckner, 2011). That said, models themselves are not without their limitations. For example, Smith et al. (2016) note that associative models based mainly on reinforcement principles, and cognitive models based mainly on uncertainty responses, are mathematically the same, and that unless one wishes to invoke Morgan’s canon, there is little reason to accept one over the other. We take a different view that perhaps the reason these models are mathematically identical is because the processes underlying them are not as different as we think; surely uncertainty monitoring is intimately tied to not only our recent but also our remote reinforcement history. As Crystal (2011, p. 417) states “if an uncertainty response was never reinforced, it seems unlikely that it would be produced by the subject, and it seems virtually impossible that it would be used functionally to express uncertainty or escape a difficult trial.”

## Concluding Comments

We have reviewed a number of studies, and we hopefully have convinced the reader that in situations where one species outperforms another the reason can often be traced to contextual variables. Macphail (1985) concluded that he did not overestimate the importance of contextual variables, and more than three decades later we would agree that contextual variables do underlie many of the differences in performance seen across species. In a companion paper (Scarf and Colombo, 2020), we have also shown that the similarities extend not only to performance on a task, but also the signatures that underlie successful performance on a task. Taken together, we fully support Macphail’s view that there are at least no qualitative differences across vertebrate species, and certainly none between birds and monkeys. On the other hand, we think there is ample support for the view that there are quantitative differences across species. Perhaps by perceiving the world through a quantitative lens of differences of degree, we can better tackle the divide between associative processes and cognitive processes.

## Author Contributions

MC and DS conceptualized and wrote the manuscript. All authors contributed to the article and approved the submitted version.

### Conflict of Interest

The authors declare that the research was conducted in the absence of any commercial or financial relationships that could be construed as a potential conflict of interest.
